# Chronic Stress Induces a Hyporeactivity of the Autonomic Nervous System in Response to Acute Mental Stressor and Impairs Cognitive Performance in Business Executives

**DOI:** 10.1371/journal.pone.0119025

**Published:** 2015-03-25

**Authors:** Renata Roland Teixeira, Miguel Mauricio Díaz, Tatiane Vanessa da Silva Santos, Jean Tofoles Martins Bernardes, Leonardo Gomes Peixoto, Olga Lucia Bocanegra, Morun Bernardino Neto, Foued Salmen Espindola

**Affiliations:** 1 Federal University of Uberlandia, Institute of Genetics and Biochemistry, Uberlandia, Brazil; 2 University of Sao Paulo, Department of Basic and Ambiental Sciences, Lorena, SP, Brazil; Max Planck Institute of Psychiatry, GERMANY

## Abstract

The present study examined the incidence of chronic stress in business executives (109 subjects: 75 male and 34 female) and its relationship with cortisol levels, cognitive performance, and autonomic nervous system (ANS) reactivity after an acute mental stressor. Blood samples were collected from the subjects to measure cortisol concentration. After the sample collection, the subjects completed the Lipp Inventory of Stress Symptoms for Adults and the Stroop Color-Word Test to evaluate stress and cognitive performance levels, respectively. Saliva samples were collected prior to, immediately after, and five minutes after the test. The results revealed that 90.1% of the stressed subjects experienced stress phases that are considered chronic stress. At rest, the subjects with chronic stress showed higher cortisol levels, and no gender differences were observed. No differences were found between the stressed and non-stressed subjects regarding salivary amylase activity prior to test. Chronic stress also impaired performance on the Stroop test, which revealed higher rates of error and longer reaction times in the incongruent stimulus task independently of gender. For the congruent stimulus task of the Stroop test, the stressed males presented a higher rate of errors than the non-stressed males and a longer reaction time than the stressed females. After the acute mental stressor, the non-stressed male group showed an increase in salivary alpha-amylase activity, which returned to the initial values five minutes after the test; this ANS reactivity was not observed in the chronically stressed male subjects. The ANS responses of the non-stressed vs stressed female groups were not different prior to or after the Stroop test. This study is the first to demonstrate a blunted reactivity of the ANS when male subjects with chronic psychological stress were subjected to an acute mental stressor, and this change could contribute to impairments in cognitive performance.

## Introduction

The incidence of chronic stress in business executives, managers and chief executive officers (CEOs) is particularly prevalent and demands various coping strategies for handling tension, anxiety, depression and even hostility [1–4]. This condition can lead to several mental and physical health issues reduce employee effectiveness and consequently affect organizational performance [5, 6]. The adaptive stress response involves vagal withdrawal, which leads to an increase in heart rate, and indicates preparedness to respond to the stressor [7]. If this response is insufficient, the autonomic nervous system (ANS) and hypothalamic-pituitary-adrenal (HPA) axis are activated [8–11]. These responses alter circulation, metabolism, learning and memory allowing the individuals to react to environmental demands [12, 13].

Catecholamines are known to control the initial response to stress, whereas glucocorticoids take a longer time to be secreted and have considerable long-lasting effects [14]. Acute stress induces specific patterns of neuronal activation within the brain and stimulates excess transmission in the dopaminergic projections in the prefrontal and anterior cingulate cortices [15, 16]. Furthermore, acute stress induces the release of higher concentrations of glucocorticoids disrupting memory formation and impairing learning [17–19] because glucocorticoids can modulate these processes [20, 21]. However, glucocorticoids can exert suppressive and permissive effects on the initial action of catecholamines [22]. Thus, higher cognitive functions that depend on prefrontal integrity, such as working memory, executive functions or selective attention, are sensitive to disturbances by acute stress [23–26]. The recurrent and uncontrolled activation of the HPA axis and the ANS (as found in chronic stress) can lead to the development of a series of pathological conditions ranging from insomnia and hypertension to fatigue and heart disease [14, 27–29].

Relevant studies about stress have focused on the response of either the HPA axis or the ANS to a stimulus that induces stress; the HPA axis is evaluated via cortisol levels, which indicate perceived stress over time, and the ANS is evaluated using salivary alpha-amylase (sAA) or catecholamines levels, which indicate acute arousal [30]. Along the same lines, we wondered how acute stress stimuli affect individuals who already have long-term HPA activation, i.e., people with chronic stressed. A few studies evaluated the HPA axis response to an acute psychosocial stressor in chronically stressed healthy subjects; these studies demonstrated a hyporeactivity of this system, which is an abnormal physiological stress response [31–33]. However, cognition and the ANS response were not evaluated. To the best of our knowledge, this study is the first to measure the sAA activity in response to an acute stressor in chronically stressed healthy subjects.

The aim of this research was to investigate the incidence of stress in male and female business executives and the influence of chronic stress in cognitive performance. To evaluate the relationship between long-term HPA activation and the reactivity of the ANS, the sAA activity was measured in response to an acute mental stressor. The following hypotheses were proposed: (1) Chronically stressed business executives have higher levels of cortisol, and this level is in accordance with the results of a psychological test; (2) chronic stress hyperactivates the HPA axis but does not change the ANS system at rest; and (3) individuals experiencing chronic stress who are subjected to an acute stressor have a blunted ANS response, leading to decreased cognitive performance.

## Materials and Methods

### Subjects

Business executives (191 subjects) from a national company with businesses in information technology, telecommunications and entertainment were subjected to an initial screening based on clinical and laboratory examinations. The subjects were recruited during the annual assessment of health held by their company. In accordance with the inclusion criteria of this research, only healthy non-smokers subjects with no history of oral diseases and those who were not under regular or incidental medication were included in the study; only the women in the luteal phase of menstrual cycle were included in this study. The final group that participated in the study consisted of 109 subjects: 75 men and 34 women aged 42.75 ± 9.50 years. The participants were instructed to avoid vigorous physical activity and refrain from consuming alcohol and caffeinated beverages for 24 h prior to the study. The subjects were given informational briefings, and they provided voluntary, written informed consent for participation. All experimental procedures were approved by the Institutional Review Board of the Federal University of Uberlandia and adhered to the Declaration of Helsinki.

### Experimental Design

**Fig 1:**
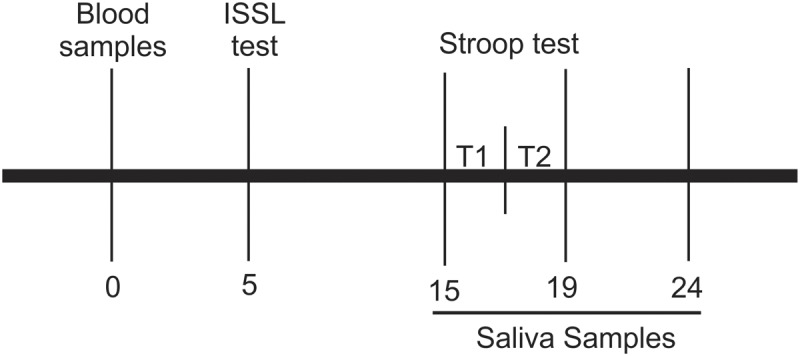
Experimental design. Numbers indicate time in minutes after the collection of blood sample. Stroop Color-Word Test is divided in two tasks, T1: Stroop Color task and T2: Stroop Color-Word task. ISSL test: Lipp Inventory of Stress Symptoms.

Blood samples were collected from all subjects to determine the cortisol concentration at rest. After the sample collection, the subjects completed the Lipp Inventory of Stress Symptoms for Adults (ISSL) and performed the two tasks of the Stroop Color-Word Test (see below). Saliva samples were collected prior to, immediately after and five minutes after the Stroop test. The experimental design is shown in Fig. 1.

### Collection of Samples

Blood from the antecubital vein (~2mL) was collected from the subjects and placed in EDTA-coated tubes by a qualified phlebotomist using standardized venipuncture techniques. All collection procedures took place at 08:30 h after 30 minutes of resting and were performed on subjects who had fasted (8 hours). The blood samples were analyzed immediately after the collection. None of the subjects reported that the blood sample collection was stressful. Samples of whole saliva were collected by having the subjects chew on a sterile cotton swab (Salimetrics, State College, PA, USA) at a frequency of 60/60 s. Saliva samples were frozen at -80°C until analysis. All collection procedures were performed between 8:30 h and 9:30 h.

### Lipp Inventory of Stress Symptoms for Adults

To evaluate the incidence of psychological stress, the subjects completed the ISSL. The Lipp Inventory is a 10-minute questionnaire based on the model of stress phases developed by Hans Seyle [34]. It is validated for people 15 years of age and older; and has been used in stress research and clinical work [35–38]. The inventory is based on the symptomatology of the stress experienced by the subjects; the symptoms vary depending to the stress phase. The inventory comprises three boxes in which the subject selects the symptoms experienced at specific times. In the first box, there are 15 physical and psychological symptoms that the subject may have experienced in the last 24 hours. The second box has symptoms related to the last week, and the third box has symptoms that the subjects could have experienced in the last month. The ISSL presents 37 physical and 19 psychological symptoms that vary in intensity and severity over time. The ISSL score is based on the symptoms and the duration during which the symptoms are experienced; the score classifies the subjects as non-stressed or stressed. The stressed group is then classified according to stress phase (alarm, resistance, pre-exhaustion and exhaustion). The inventories were applied, corrected and interpreted by trained psychologists according to the inventory’s handbook.

For our research, the subjects were divided into non-stressed (NS) and stressed (S) groups based on the ISSL score. The stressed group comprised the subjects in the resistance and pre-exhaustion phase. The subjects in the alarm phase were excluded from the stressed group because this phase does not reflect chronic exposure to adverse psychological stressors.

### The Stroop Color-Word Test

The Stroop Color-Word Test has been used extensively in psychophysiological research. In this study, the test was used to assess cognitive performance and as an acute mental stressor because previous studies have shown that the test induces mental stress, as indicated by increased cortisol and catecholamines levels and increased heart rate [39–41]. The test was conducted between 8:30 h and 9:30 h in a sound-attenuated, electrically shielded room at the facilities of the company. The Stroop Color-Word Test assesses the subjects’ selective attention and reaction time. The test is divided into two tasks, namely “Color” and “Color-Word”. The Stroop Test consists of a sheet of 112 printed color names (red, green, blue, yellow) arranged in four columns of 28 names each. The names are printed in one of four different colors of ink (red, green, blue or yellow), but the color name is not printed in a matching color (e.g., the name RED is never printed in red ink) [42]. In the Color task, the subjects were required to read the words aloud as quickly as possible regardless of the color of ink in which the words are printed. In the Color-Word task, the subjects were instructed to identify the color in which the stimuli were printed and to do so as quickly and accurately as possible. The reaction time was measured in seconds from the onset of the stimulus, and the accuracy of each response was checked. The examiners did not point out the errors made during the test.

### Cortisol

Blood samples were centrifuged at 3000xG for 5 minutes at 4°C after the collection. The plasmatic cortisol concentration was determined using an immunoassay (Cobas e411 analyzer-Roche Diagnostics) with kits obtained from Roche Diagnostics. The intra-assay coefficient of variation for duplicate samples was 3.2%.

### Salivary Alpha-Amylase Activity

On the day of analysis, the saliva samples were thawed and centrifuged at 1500xG for 15 minutes at 4°C to remove mucins. The saliva samples were diluted (1:200) in MES buffer (50 mM MES, 300 mM NaCl, 5 mM CaCl_2_ and 140 mM KSCN; pH 6.3), and 8 μl was pipetted into a microplate; then, 320 μl of pre-heated (37°C) substrate solution (2-chloro-4-nitrophenyl-β-D-galactopyranosylmaltoside: GALG2-CNP) was added. The optical density was read at 405 nm in one-min intervals for three minutes at 37°C using a microplate reader. The enzyme activity (U/mL) was determined using the following formula: [Absorbance difference per minute × total assay volume (328 μl) × dilution factor (200)]/ [millimolar absorptivity of 2-chloro-4-nitrophenol (12.90) × sample volume (8 μl) × light path (.97)] [43]. The enzyme activity (U/mL) was then multiplied by the flow rate (mL/min) to estimate the sAA secretion rate (U/mL/min). All reagents were purchased from Sigma-Aldrich (Saint Louis, Missouri, USA). The intra-assay coefficient of variation for duplicate samples was below 5%.

### Statistical Analysis

The results are expressed as the means ± standard error (SE). The data were tested for normality using the Kolmogorov-Smirnov test prior to analysis. To compare the results of the laboratory exams between males and females the unpaired t test was performed. All of the others results were analyzed using a one-way analysis of variance (ANOVA) followed by the Tukey test. To demonstrate the effects of chronic stress (NS vs S) and the acute mental stressor (Stroop test) on the sAA results, a two-way factorial ANOVA was performed, p ≤ 0.05 was considered significant.

## Results

### Characterization of the Subjects

The 109 subjects (75 men and 34 women) who participated in the study were subjected to laboratory exams. All of the participants were healthy because the results were within the reference values, and some gender differences were observed. The male subjects presented higher levels of triglycerides, low density lipoprotein (LDL), creatinine and uric acid and lower levels of high density lipoprotein (HDL) compared with those of the female subjects [Triglycerides: Male = 111.7 ± 8.5 mg/dL, Female = 71.4 ± 8.2 mg/dL (p = 0.002); LDL: Male = 111.1 ± 6.6 mg/dL, Female = 94.7 ± 5.0 mg/dL (p = 0.05); Creatinine: Male = 1.0 ± 0.03 mg/dL, Female = 0.8 ± 0.03 mg/dL (p < 0.0001); Uric acid: Male = 5.7 ± 0.2 mg/dL, Female = 3.5 ± 0.2 mg/dL (p < 0.0001); HDL: Male = 43.8 ± 2.2 mg/dL, Female = 59.0 ± 2.9 mg/dL (p = 0.0003)] (Table 1).

**Table 1:** Clinical and Laboratory information of the subjects.

	Males Mean ± SE	Females Mean ± SE
Body Mass Index	23.9 ± 0.6 Kg/m^2^	23.1 ± 0.4 Kg/m^2^
Sistolic Pressure	113.8 ± 2.1 mmHg	108.6 ± 2.3 mmHg
Diastolic Pressure	75.4 ± 1.4 mmHg	71.4 ± 1.4 mmHg
Cardiac Frequency	66.2 ± 1.0 bpm	68.9 ± 1.5 bpm
Triglycerides	111.7 ± 8.5 mg/dL	71.4 ± 8.2 mg/dL (*)
Cholesterol	176.2 ± 7.5 mg/dL	167.2 ± 5.7 mg/dL
High Density Lipoprotein (HDL)	43.8 ± 2.2 mg/dL	59.0 ± 2.9 mg/dL (*)
Low Density Lipoprotein (LDL)	111.1 ± 6.6 mg/dL	94.7 ± 5.0 mg/dL (*)
Glycemia	86.4 ± 1.6 mg/dL	83.3 ± 1.7 mg/dL
Creatinine	1.0 ± 0.03 mg/dL	0.8 ± 0.03 mg/dL (*)
Uric Acid	5.7 ± 0.2 mg/dL	3.5 ± 0.2 mg/dL (*)
Thyroid-stimulating Hormone (TSH)	1.6 ± 0.2 μUI/mL	1.6 ± 0.3 μUI/mL
Thyroxine (T4)	1.3 ± 0.05 ng/dL	1.2 ± 0.06 ng/dL
Alanine Aminotransferase (ALT)	34.4 ± 2.7 U/L	29.9 ± 2.4 U/L
Aspartate Aminotransferase (AST)	21.7 ± 1.4 U/L	18.6 ± 1.3 U/L
Gamma-Glutamyl Transferase (GGT)	26.8 ± 3.2 U/L	24.4 ± 1.6 U/L

Values are expressed as mean ± standard error (SE).

(*) Indicates significant differences, p ≤ 0.05 (unpaired t test).

### Incidence of Perceived Psychological Stress, Cortisol and Amylase Activity

The analyses of perceived stress indicated that 46.8% of the subjects, 34 male and 17 female, experienced significant symptoms associated with any of the phases of stress (Table 2) and that 90.1% of them, 31 male and 15 female, were in the resistance and pre-exhaustion phases, which are characterized as chronic stress (Table 3). The subjects who reported some level of stress revealed substantially more psychological than physical symptoms. Based on these scores on the ISSL test, the subjects were divided into the non-stressed (NS) and stressed (S) groups, with the latter group comprising subjects in the resistance and pre-exhaustion phase. The subjects who were identified to be in the alarm phase were excluded from the cortisol and sAA analysis because the symptoms experienced in the last 24 hours did not reflect chronic exposure to adverse psychological stressors.

**Table 2:** Incidence and symptoms of stress in business executives assessed by Lipp Inventory of Stress Symptoms for Adults (ISSL).

	Non-stress (n)	Stress (n)	Total (n)	Stress Symptoms (n)		ISSL Score	ISSL Score
				Physiologic	Psychological	Non-Stress (mean ± SE)	Stress (mean ± SE)
Male	41	34	75	10	24	3.17 ± 0.34	11.50 ± 0.92
Female	17	17	34	2	15	3.59 ± 0.56	14.76 ± 2.06
Total	58	51	109	12	39	3.29 ± 0.29	12.59 ± 0.93

Values are expressed as number of subjects (n) and ISSL Scores are expressed as mean ± standard error (SE).

**Table 3:** Incidence of stress phases in business executives assessed by Lipp Inventory of Stress Symptoms for Adults (ISSL) and gender differences.

	Stress Phases (n)			
	Alarm	Resistance	Pre-exhaustion	Total
Male	3	29	2	34
Female	2	11	4	17
Total	5	40	6	51

Values are expressed as number of subjects (n).

**Fig 2:**
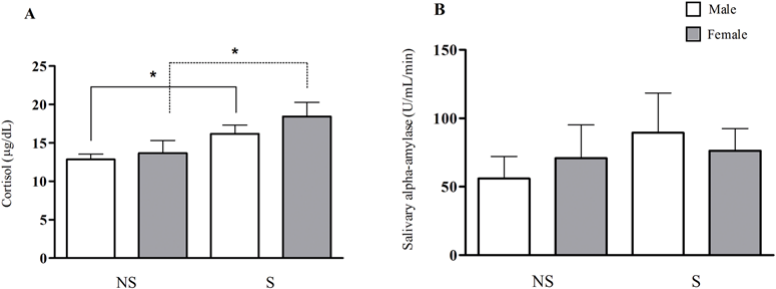
Biological variables in non-stressed (NS) and stressed (S) subjects. **A.** Serum cortisol in non-stressed and stressed male and female subjects at rest. **B.** Salivary alpha-amylase activity prior to the cognitive task in non-stressed and stressed male and female groups. (*) Indicates significant differences, p ≤ 0.05 (One-way ANOVA).

The chronically stressed subjects showed higher levels of cortisol at rest compared with those of the non-stressed group; this effect was independent of gender [NS/Male = 12.8 ± 0.7 μg/dL; S/Male = 16.2 ± 1.1 μg/dL; NS/Female = 13.7 ± 1.7 μg/dL; S/Female = 18.4 ± 1.8 μg/dL (p ≤ 0.05)]. However, prior to the Stroop test, no difference regarding sAA activity was observed between the groups [NS/Male = 55.9 ± 16.2 U/mL/min; S/Male = 89.4 ± 29.0 U/mL/min; NS/Female = 70.8 ± 24.2 U/mL/min; S/Female = 76.2 ± 16.2 U/mL/min] (Fig. 2).

### Influence of Chronic Psychological Stress on Cognitive Performance

**Fig 3:**
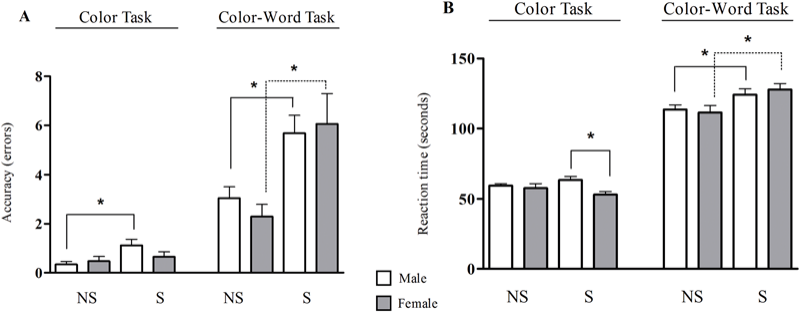
Cognitive performance of non-stressed (NS) and stressed (S) subjects during the Stroop test. **A.** Accuracy of response in Color task and Color-Word task of Stroop test in male and female subjects. **B.** Reaction time in both tasks of the Stroop test in male and female subjects. (*) Indicates significant differences, p ≤ 0.05 (One-way ANOVA).

During the two tasks of the Stroop test, the reaction time and number of errors were assessed, and chronic stress significantly impaired the quality of response. The male stressed group committed more errors during the congruent stimulus (Color Task) than did the non-stressed male group [NS/Male = 0.3 ± 0.1 errors; S/Male = 1.1 ± 0.3 errors (p ≤ 0.05)], and no difference was observed between the non-stressed and stressed female groups [NS/Female = 0.5 ± 0.2 errors; S/Female = 0.6 ± 0.2 errors]. Regarding the incongruent stimulus (Color-Word task), chronic stress had the same influence in the male and female groups; it increased the rate of errors [NS/Male = 3.0 ± 0.5 errors; S/Male = 5.7 ± 0.7 errors; NS/Female = 2.3 ± 0.5 errors; S/Female = 6.1 ± 1.2 errors (p ≤ 0.05)]. Regarding the reaction time in the congruent stimulus, the stressed male group presented a longer reaction time than the stressed female group [S/Male = 63.6 ± 2.4 sec; S/Female = 53.2 ± 2.2 sec (p ≤ 0.05)]. In the Color-Word task, chronic stress increased the reaction time in both genders [NS/Male = 113.7 ± 3.2 sec; S/Male = 124.1 ± 4.3 sec; NS/Female = 111.5 ± 5.0 sec; S/Female = 127.8 ± 4.4 sec (p ≤ 0.05)] (Fig. 3).

### The Effect of an Acute Mental Stressor on the Reactivity of the ANS in Chronically Stressed Subjects

**Fig 4:**
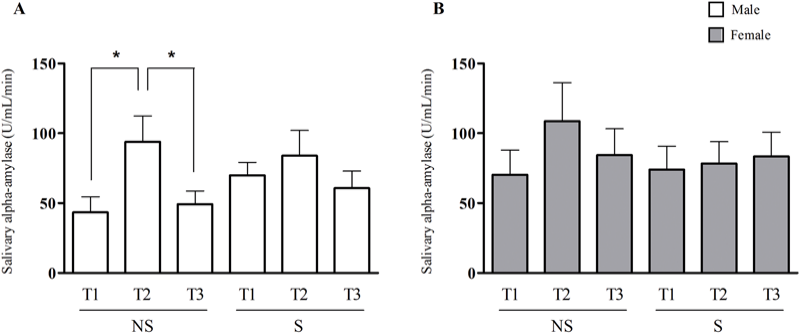
Salivary alpha-amylase activity during an acute mental stress test in non-stressed (NS) and stressed (S) subjects. **A.** Salivary alpha-amylase activity assessed prior to the Stroop test (T1), immediately after the Stroop test (T2) and five minutes after the test (T3) in non-stressed and stressed males subjects. **B.** Salivary alpha-amylase activity assessed at T1, T2 and T3 in non-stressed and stressed females subjects. (*) Indicates significant differences, p ≤ 0.05 (One-way ANOVA).

After the Stroop test, the non-stressed male group showed an increase in sAA activity, and the sAA activity decreased five minutes after the end of the test [NS/Male: T1 = 43.5 ± 11.2 U/mL/min; T2 = 94.0 ± 18.4 U/mL/min, T3 = 49.3 ± 9.4 U/mL/min (p ≤ 0.05)]. In the stressed male group, this reactivity of the ANS was not observed because there was no difference in the sAA activity prior to vs after the acute mental stressor [S/Male: T1 = 69.9 ± 9.3 U/mL/min; T2 = 84.0 ± 18.0 U/mL/min; T3 = 60.6 ± 12.3 U/mL/min]. No difference was observed for both the stressed and non-stressed female groups [NS/Female: T1 = 70.3 ± 17.7 U/mL/min; T2 = 108.6 ± 27.5 U/mL/min, T3 = 84.5 ± 18.7 U/mL/min and S/Female: T1 = 74.1 ± 16.6 U/mL/min; T2 = 78.3 ± 15.7 U/mL/min, T3 = 83.4 ± 17.4 U/mL/min] (Fig. 4). The two-way factorial ANOVA analysis demonstrated that the interaction between the chronic stress and the acute mental stressor have significant effect on the sAA activity (F (2,156) = 3.45 p = 0.034).

## Discussion

In our study, we selected a group of subjects who were already experiencing chronic psychological stress conditions and who had higher levels of cortisol. We investigated the response of the ANS to an acute stressor and the cognitive performance of these subjects compared with that of a non-stressed group. To our knowledge, our study is the first to assess sAA activity in response to an acute stressor in chronically stressed healthy subjects; it was also the first to describe a blunted reactivity of the ANS in chronically stressed males who were subjected to an acute mental stressor. The results indicated that in the chronically stressed males, the hyperactivation of the HPA axis appears to lead to a hyporeactivity of the ANS in an acute stress situation and that this physiological situation affects cognitive performance.

This result was consistent with our expectation that the higher concentration of cortisol at rest in the stressed group suggests a hyperactivation of the HPA axis. The glucocorticoids released from the adrenal cortex are required to resist the stressor but are also responsible for the pathological changes that are characteristic of the almost-exhaustion phase [44]. Consistent with this hypothesis, other studies have found similar increases in cortisol in subjects with an overcommitment to work [32, 45] and a lack of control at work [46]. Physiologically rather than psychologically adverse conditions, such as asthma [47], rheumatoid arthritis [48] and atopic dermatitis [49], also lead to variations in the concentration of cortisol at rest; however, all of the subjects in this study were healthy thus this increased in cortisol levels is due to the psychological chronic stress.

Variations in sAA activity in response to acute stressors are well-accepted as a surrogate marker of autonomic activity [43, 50]. In our study, prior to the Stroop test, no difference regarding sAA activity was observed between the groups. An explanation for this result is that salivary alpha-amylase activity indicates acute arousal [30], thus without an acute stimulus no difference between groups were found in the ANS response. The increase in sAA activity was observed after an acute mental stressor only in healthy male subjects who were not previously chronically stressed. These results corroborate those of previous studies that also observed an increase in the sAA activity in healthy non-stressed subjects after acute stressors, such as surgery video viewing [51], arithmetic tasks [52], memory test [53] and skydiving [54].

However, we showed that this increase in sAA activity after an acute mental stressor was not observed in healthy chronically stressed male subjects. Most studies thus far have either assessed the kinetics of cortisol and sAA in response to a broad range of stressors or compared their dynamics between subjects suffering from chronic stress associated with diseases [55–60]. Although only three studies evaluated the HPA axis response to an acute psychological stressor in chronically stressed healthy subjects. In chronic work stressed middle managers (in terms of effort-reward-imbalance (ERI) model) subjected to the Stroop Color-word conflict task a reduced maximal cortisol responses was observed, providing preliminary evidence for attenuated neuroendocrine stress responses [31]. More recently, lower salivary cortisol and norepinephrine responses were reported after the Trier Social Stress Test (TSST) in higher overcommitment (OC) male adults [32]. Furthermore, a hyporeactivity of the HPA axis was also described after an acute psychosocial stressor in chronically stressed healthy schoolteachers (according to the ERI/OC model) [33]. However, none of these studies have evaluated the ANS response and cognition. To our knowledge, this study is the first to assess sAA activity in response to an acute stressor in chronically stressed healthy subjects; and it is also the first to describe a blunted reactivity of the ANS in chronically stressed males who were subjected to an acute mental stressor.

Animal studies have investigated the effects of glucocorticoids and catecholamines in response to stress [22]. As previously mentioned, these studies indicated that glucocorticoids suppress the early effects of catecholamines in the brain and thus impair memory formation and learning after repeated exposure to stress [22, 61–63]. However, not all human studies on cortisol and sAA support this notion. An asymmetry between the HPA axis and the ANS was described, and no correlation between cortisol and sAA was observed in maltreated youth [57]. Furthermore, lower concentrations of cortisol and higher sAA secretion rates were linked to a positive effect and approach behavior in toddlers [64]. In contrast, evidence of a symmetrical interaction between the HPA axis and the ANS was reported in children who externalized and internalized problems; these children showed higher levels of cortisol, skin conductance and sAA secretion rates [65]. An association between low concentrations of cortisol and low sAA secretion rates also predicted aggressive behavior in young men [66]. Our data add further evidence of an asymmetry between the HPA axis and the ANS in chronically stressed males; an elevated concentration of cortisol appears to lead to desensitization to catecholamines.

The role of catecholamines and elevated concentrations of cortisol in memory formation and learning have been previously demonstrated in human subjects. Beta-adrenergic blockage, for instance, impairs the formation of memories of emotionally arousing stories [67], whereas the administration of a beta-adrenergic agonist enhances memory consolidation [68, 69]. Moreover, subjects treated with prednisone had lower performance scores on tests of explicit memory [70] and delayed word recall [71] compared with those of drug-free controls. The mechanisms behind the results of this appear to be the disruption of synaptic plasticity and the blunting of hippocampal excitability due to an elevated concentration of glucocorticoids [22, 72–74].

The HPA axis and the ANS show markedly different responses to acute stress [60, 75, 76]. Here, we show that males who were exposed to chronic stress obtained inferior performance scores with a lower reactivity in the ANS during an acute mental stressor. This finding suggests an interaction between the responses of the HPA axis and the ANS to stress although the responses show opposite dynamics (higher cortisol vs. lower sAA). A higher concentration of cortisol may induce alterations in the sensitivity to catecholamines in rats [77–79]. Thus, long-term exposure to life stress may result in a lower reactivity of the ANS; however, this possibly is speculative.

In our study, subjects with higher concentrations of cortisol and higher scores of perceived stress showed lower scores during the Stroop test independently of the gender. A considerable body of research has also demonstrated that elevated concentrations of cortisol have negative effects on cognitive function and memory consolidation [40, 80–82]. The administration of a single dose of either hydrocortisone or placebo to healthy subjects prior to the Stroop test resulted in an inferior score for those who received the drug [62]. A possible mechanism for such alterations could be that long-term exposure to glucocorticoids modifies the gene expression in the brain areas involved in learning, behavior, language and memory [61, 83–85].

In conclusion, chronic psychological stress associated with higher levels of cortisol impairs cognitive performance in business executives independently of genders, and a blunted reactivity of the ANS was observed when chronic stressed males were subjected to an acute mental stressor. Considering our results, we argue that over time, elevated concentrations of cortisol may impair the regular action of catecholamines in response to stress, and these events could contribute to impairments in cognitive performance.

## Limitations

No difference was observed between the non-stressed and stressed female groups regarding the response of the ANS to an acute stressor, and females present fewer cognitive losses; although, this study cannot conclude that chronic stress affects females less than males due to the small number of female subjects in the study and the variety of values obtained in the analysis of the samples of this group. These represent limitations of this study. Other studies should be performed with chronically stressed females (including those at different points in the menstrual cycle) to establish the relevant physiological and cognitive responses to an acute stressor.
